# Intracerebral atypical calcification in nongalenic pial arteriovenous fistula: a case report

**DOI:** 10.1186/1757-1626-1-335

**Published:** 2008-11-19

**Authors:** Seyed Ali Fakhr Tabatabai, Mehdi Zeinali Zadeh, Zohreh Habibi, Ali Tayebi Meybodi, Mohammad Hashemi

**Affiliations:** 1 Department of Neurosurgery, Imam Khomeini hospital, Tehran University of Medical Sciences, Tehran, Iran

## Abstract

Nongalenic intradural arteriovenous fistulas, although uncommon, are clinically important. Choosing the appropriate therapeutic approach has been a controversial issue within the last decade.

A 15-year-old male was presented with a calcified nongalenic arteriovenous fistula in the left parietal region, supplied by the left middle cerebral artery, and draining into the left lateral sinus. The patient was managed surgically with traditional clipping the feeder artery, along with piecemeal resection of the huge calcified mass. Although endovascular methods may be the treatments of choice in similar cases, in such huge calcified lesion, non-amenable to endovascular occlusion, open surgery seems to be preferred.

## Background

Arteriovenous fistula (AVF) is a rare entity occurring at different sites of the intracranial structures. The lesion has been described as a direct connection between an artery and a vein without any intervening nidus, dissimilar to true arteriovenous malformation (AVM) [1]. These lesions are defined as dural or intradural AVFs [2]. Intradural cerebral fistulas, the so-called pial AVFs, comprise 1.6% of all Childhood arteriovenous malformations [3], with the majority of the lesions involving the vein of Galen and a smaller proportion are nongalenic cerebral AVF [4].

In this report, a case of huge nongalenic AVF with a pattern of wonderful intracerebral calcification is presented.

## Case report

A 15-year-old left handed male was admitted because of a 5-year history of weakness in the right extremities, aggravated within 3 months. The patient was a product of normal gestation and delivery, and other than a history of birth head trauma, he had uneventful medical history and perfect developmental milestones.

At admission time, physical examination revealed a right hyperreflexia, hemihyposthesia and hemiparesis, with the estimated force of 4/5 according to Louisiana State University Medical Center grading system [5].

**Figure 1:**
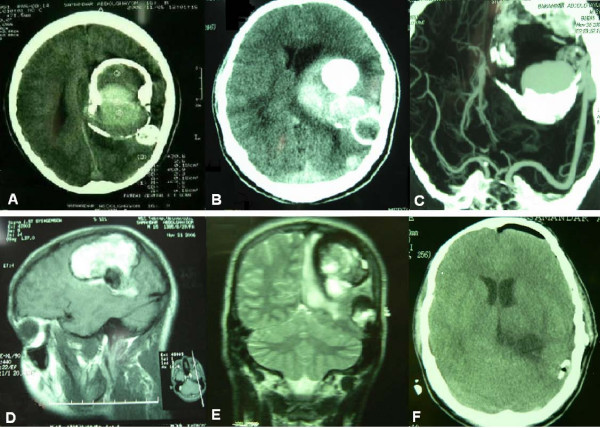
**Noncontrast computed tomography showing a huge calcified egg-shaped mass**. With a small adjacent calcified lesion (A) and a new episode of hemorrhage (B). CT-Angiography of the lesion (C). T1-weighted parasagittal (D) and T2-weighted coronal (E) magnetic resonance images reveal large mass lesion with signal void rim indicative of calcification and central hypersignal intensity due to met-hemoglobin deposition. Post-operative brain CT scan (F).

**Figure 2:**
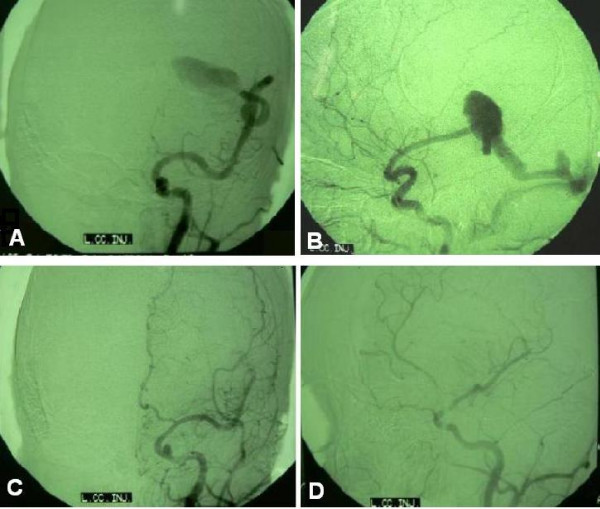
**Digital subtraction angiogram:** Early arterial phase of the left internal carotid injection revealing an intradural nongalenic arteriovenous fistula supplied from the left MCA and diminished flow in the left ACA due to the high-flow fistula (A). The oblique view of the late arterial phase showing the varix draining into the left lateral sinus (B). Post-operative left internal carotid angiogram revealing the left ACA filling in normal pattern, and the left MCA re-filling to the point that the lesion was previously resected (C and D).

Brain computed tomography (CT) and magnetic resonance imaging (MRI) disclosed a huge (7.8 × 5.3 cm) intra-axial elliptical mass with a calcified margin in the left parietal region, causing a midline shift of 1 centimeter and containing hemorrhagic components of different ages (Figure 1). Four-vessel brain digital subtraction angiography (DSA) revealed an intradural nongalenic AVF and an associated varix, supplied from the left middle cerebral artery (MCA) and drained to the left lateral sinus just adjacent to the Torcular Herophili (Figure 2A and 2B). The left anterior cerebral artery (ACA) was not completely filled after dye injection, owing to high flow through the fistula.

Echocardiography displayed no heart failure or valvular abnormality.

### Surgery

**Figure 3:**
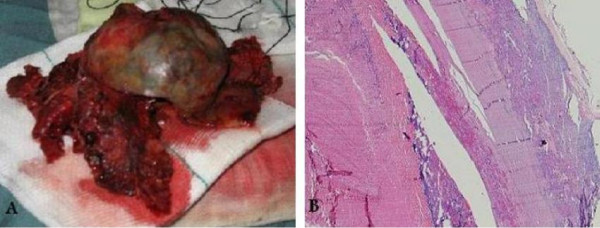
**Gross appearance of the resected calcified lesion **(A). Photomicrograph of the lesion (B) showing vascular structures with atheromatous plaques. H & E, original magnification ×10.

Open microsurgery was performed, and through opening the Sylvian fissure the hypertrophic distal segment of MCA was exposed, reaching to a calcified mass in the distal part of the Sylvian fissure. The feeder artery was clipped proximal to its entrance into the mass. Piecemeal resection of the calcified wall (Figure 3-A) was performed following the evacuation of hematoma inside the lesion. The venous outflow was ligated beside the lateral sinus, along with removing the adjacent varix.

The patient was sedated for 2 days using midazolam and sodium thiopental. He was conscious following extubation on the third day without neurological deterioration. Control brain CT on the day following the surgery revealed neither remnants nor hematoma (figure 1-F). Control angiography performed on the 20^th ^day displayed the left ACA to fill in a normal pattern, with the MCA stem refilling to the end of Sylvian fissure, where the lesion was previously resected (Figure 2-C and 2D).

Histopathological assessments demonstrated mature vascular structures with clot and atheromatous plaques and encircling calcified layer (Figure 3-B).

The patient returned to school one month later, and no episode of headache or seizure has happened in 2 years follow-up.

## Discussion

Arteriovenous fistula is an uncommon but clinically important condition. Theoretically, the fistula consists of an arteriovenous shunt, having a direct passage with no intervening nidus or vessels [6,7].

Various kinds of classification of AVFs have been established, based on location, draining veins, flow speed, and anatomical structures. Basically, arteriovenous fistulas are categorized to dural and intracerebral lesions. The latter is equivalent to the so-called intra-axial, intradural, cerebral, and pial AVFs. Pial fistulas differ from the dural type as they acquire feeders from cortical arteries. They are further categorized into galenic and nongalenic forms. Nongalenic cerebral AVFs do not involve the persistent embryonic median prosencephalic vein [3,1]. Nongalenic AV fistula was first described by Dandy who treated it by ligation of the feeding artery through an open surgical procedure [8]. However, regarding to the infrequency of nongalenic AVFs, they were grouped with AVMs for several decades until angiography commenced as a diagnostic modality to define the lesion more exactly.

Although AVFs may present in childhood, most cases remain undetected until later life.

These entities usually manifest in adulthood with hemorrhage, headache, seizure, elevated venous pressure, mass effect, or cerebral ischemia due to steal phenomenon [2,6]. The most common presentations in pediatric group are cardiac insufficiency, epilepsy, and macrocrania in descending order [9]. If remain untreated, it can result in subependymal or cortical atrophy, white mater calcification and delayed myelinization [7].

At present, four-vessel cerebral angiography is the gold standard diagnostic procedure which can distinguish nongalenic AVFs from AVMs [1]. Angiographic diagnostic criteria for AVFs consist of; (1) rapid circulation time because of high-velocity flow, (2) enlarged feeding artery, and (3) direct filling of a large varix [10].

Obliteration of the fistula through standard craniotomy, first described by Dandy in 1928, is still advocated by many surgeons. However, for deep seated lesions or fistulas in eloquent regions less invasive endovascular alternatives have been considered recently.

### This case

In this patient, there are several hints suggesting the fistula to be originated from early childhood or even prenatal period. Although head injury is more associated with dural rather than nongalenic fistula, birth time head trauma may be proposed as a potential predisposing factor in this patient. Left-handedness can imply a potential damage to dominant hemisphere within the first stages of the patient's life, as regards the hypotheses advocating the role of early brain damage in pathological left hand domination [11].

Calcification which is more probable in pial than dural AV lesions, should be considered too [12]. The characteristics of curvilinear calcification in the corticomedullary junction and at the bottom of cerebral gyrus in the cases of dural AVF are different from that of pial fistula [12]. The mechanism of calcification accompanied with pial AVFs is a dystrophic process due to hypoperfusion caused by steal phenomenon or venous congestion over a long time [12,13]. The thick mural calcification implies a long-standing course of cerebral damage in this patient. A case of calcification around a pial AVF has been already reported by Oda et al in the background of Rendu-Osler-Weber disease [4]. However, such huge calcified covering in the setting of sporadic pial AVF seems to be unprecedented.

Considering the calcified mass accompanied by significant midline shift in this case, even if the fistula had been obliterated by endovascular methods alleviating the mass effect would have been impossible. Indeed, the overall risk of endovascular occlusion of AVF in one stage and surgical removal of the calcified mass in another stage would outweigh the risk of a single operation for occluding the accessible feeder artery and removing the mass simultaneously. The calcified shell would physically restrict approach and handling of endovascular devices.

Hence, in spite of concerns about the potential drawbacks of different therapies, the most appropriate options should be tailored for each patient considering particular situations.

## Conclusion

Various therapeutic options have been proposed for nongalenic AVFs. Although endovascular techniques have been shown to be less invasive, such massive calcified lesions which make significant mass effect or are unlikely to dwindle after transarterial occlusion are better managed surgically.

## Consent

Written informed consent was obtained from the patient and his parents for publication of this case report and accompanying images. A copy of the written consent is available for review by the Editor-in-Chief of this journal.

## Competing interests

The authors declare that they have no competing interests.

## Authors' contributions

SAFT performed the microsurgery and gave the final approval for the version to be submitted.

MZZ made contribution to conception and analyzed the patient data.

ZH made contribution in collecting data and drafting the manuscript.

ATM was contributor in revising the manuscript.

MH was contributor in drafting the manuscript.
